# Influence of Camphor on the Sexual Organs

**Published:** 1844-01-27

**Authors:** 


INFLUENCE OF CAMPHOR ON THE SEXUAL ORGANS.

Among the ancients, camphor was celebrated as an anti-aphrodisiac, and several of the moderns have borne testimony to its influence on the sexual organs. Alibert cites the case of a nymphomanic patient at St. Louis, whom he cured by a drachm of camphor at a dose; and Esquirol has successfully treated hysteric nymphomaniacs with the same remedy. M. Guersant speaks of a druggist whose virility failed him after the perpetual employment, *during one day*, of a smelling-bottle containing spirits of camphor; also of a young woman, of ardent and excitable temperament in whom the use of camphor pills subdued all sexual desires; and Dupuytren attended a medical student in whom impotence had ensued after having slept in a room in which camphor was kept. M. Felix Le Gros, asserts that he has found this substance the most active remedy he could use for blennorrhagic erections and chordee.—*Lancet, from Gaz. des Hôp.*

